# The influence of tag sequence on recombinant humanized collagen (rhCol) and the evaluation of rhCol on Schwann cell behaviors

**DOI:** 10.1093/rb/rbad089

**Published:** 2023-10-16

**Authors:** Mingxuan Bai, Ning Kang, Yang Xu, Jing Wang, Xinxing Shuai, Caojie Liu, Yixuan Jiang, Yu Du, Ping Gong, Hai Lin, Xingdong Zhang

**Affiliations:** State Key Laboratory of Oral Diseases & National Center for Stomatology & National Clinical Research Center for Oral Diseases, West China Hospital of Stomatology, Sichuan University, Chengdu, Sichuan 610041, P.R. China; State Key Laboratory of Oral Diseases & National Center for Stomatology & National Clinical Research Center for Oral Diseases, West China Hospital of Stomatology, Sichuan University, Chengdu, Sichuan 610041, P.R. China; National Engineering Research Center for Biomaterials, College of Biomedical Engineering, Sichuan University, Chengdu, Sichuan 610065, P.R. China; National Engineering Research Center for Biomaterials, College of Biomedical Engineering, Sichuan University, Chengdu, Sichuan 610065, P.R. China; National Engineering Research Center for Biomaterials, College of Biomedical Engineering, Sichuan University, Chengdu, Sichuan 610065, P.R. China; State Key Laboratory of Oral Diseases & National Center for Stomatology & National Clinical Research Center for Oral Diseases, West China Hospital of Stomatology, Sichuan University, Chengdu, Sichuan 610041, P.R. China; State Key Laboratory of Oral Diseases & National Center for Stomatology & National Clinical Research Center for Oral Diseases, West China Hospital of Stomatology, Sichuan University, Chengdu, Sichuan 610041, P.R. China; State Key Laboratory of Oral Diseases & National Center for Stomatology & National Clinical Research Center for Oral Diseases, West China Hospital of Stomatology, Sichuan University, Chengdu, Sichuan 610041, P.R. China; State Key Laboratory of Oral Diseases & National Center for Stomatology & National Clinical Research Center for Oral Diseases, West China Hospital of Stomatology, Sichuan University, Chengdu, Sichuan 610041, P.R. China; State Key Laboratory of Oral Diseases & National Center for Stomatology & National Clinical Research Center for Oral Diseases, West China Hospital of Stomatology, Sichuan University, Chengdu, Sichuan 610041, P.R. China; National Engineering Research Center for Biomaterials, College of Biomedical Engineering, Sichuan University, Chengdu, Sichuan 610065, P.R. China; National Engineering Research Center for Biomaterials, College of Biomedical Engineering, Sichuan University, Chengdu, Sichuan 610065, P.R. China

**Keywords:** recombinant humanized collagen, Tag sequence, peripheral nerve regeneration, Schwann cell

## Abstract

Recombinant humanized collagen (rhCol) was an extracellular matrix (ECM)-inspired biomimetic biomaterial prepared by biosynthesis technology, which was considered non-allergenic and could possibly activate tissue regeneration. The influence of tag sequence on both structures and performances of rhCol type III (rhCol III) was investigated, and the effect of rhCol III on cell behaviors was evaluated and discussed using Schwann cells (SCs) as *in vitro* model that was critical in the repair process after peripheral nerve injury. The results demonstrated that the introduction of tag sequence would influence both advanced structures and properties of rhCol III, while rhCol III regulated SCs adhesion, spreading, migration and proliferation. Also, both nerve growth factor and brain-derived neurotrophic factor increased when exposed to rhCol III. As the downstream proteins of integrin-mediated cell adhesions, phosphorylation of focal adhesion kinase and expression of vinculin was up-regulated along with the promotion of SCs adhesion and migration. The current findings contributed to a better knowledge of the interactions between rhCol III and SCs, and further offered a theoretical and experimental foundation for the development of rhCol III-based medical devices and clinical management of peripheral nerve injury.

## Graphical abstract

**Figure:**
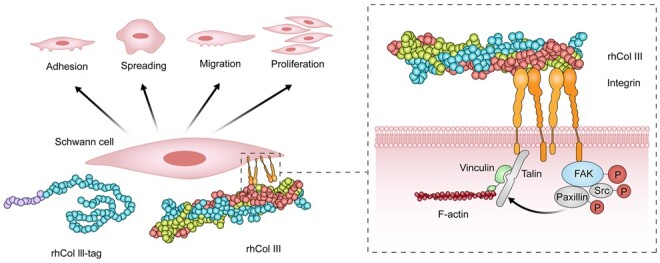

## Introduction

Collagen, as the main component of native extracellular matrix (ECM), has been widely applied to construct cell micro-environment that could modulate cell behaviors and promote tissue engineering [1]. However, the predominantly utilized collagen was an animal-derived native macromolecule that might take risks of virus contaminations and immunogenicity, especially when it was applied as the starting material for tissue engineering medical products [2, 3]. With the development of DNA recombinant and biosynthesis technologies, the design and development of recombinant collagen might be a potential strategy to overcome the difficulties confronted in the development of animal-derived collagen [2, 4]. Recombinant collagen could be prepared by editing and transcribing the human collagen gene or its fragments into host microorganisms like *Escherichia coli* and yeast, then purifying the expressed protein after a period of fermentation under optimized conditions [5]. In the previous study, a recombinant humanized collagen type III (rhCol III) was obtained from 16 tandem repeats of a functional domain (Gly483-Pro512) in human collagen type III [6], and its biosafety and functionality were confirmed by evaluating *in vitro* co-culture with cells, *in vivo* implantation *via* cutaneous injection [7], cardiovascular scaffold coating [8, 9], etc. In recent years, some studies reported rhCol could contain not only the repeats of functional domains but also linking or tag sequence, which was not encoded by native human collagen gene but artificially introduced for the capture and purification purpose of the expressed protein [10, 11]. Therefore, it was necessary to investigate the influence of tag sequence on the structure of rhCol and the interactions between rhCol and cells, especially when the advanced structures of collagen were considered critical to generate a biological response.

To understand the structure transformation of rhCol III with or without tag sequence, both computer-assisted prediction and structure characterization were effective. In recent years, programmed algorithms like AlphaFold2 (AF2) developed by DeepMind have made breakthrough progress with the help of artificial intelligence technology [12]. With the assistance of the state-of-art technology, not only an accurate prediction of three-dimensional (3D) structures of proteins could be achieved based on amino acid sequences [12] but also the structural and functional mechanisms of proteins could be further understood and verified [13]. Molecular docking offered an efficient and rapid method to predict the binding affinities of proteins based on the structures, which could provide a complementary verification for the structure characterizations [14, 15]. Thus, both molecular docking and traditional protein structure characterizations were utilized in this study to understand the relationship between structures and functions of rhCol III with and without tag sequence.

In addition, to evaluate the modulation effect of rhCol III on cell behaviors, Schwann cells (SCs) were chosen as a co-culture model in the study. As the glial cells in the peripheral nerve system, SCs played indispensable roles during neural development and regeneration [16]. Following nerve injury, axonal degeneration and myelinolysis caused SCs to dedifferentiate into a repair phenotype, contributing to the removal of axon and myelin-derived debris, the filling of the empty endoneurial tube, and the formation of Büngner bands. Neurotrophic factors were released by SCs in Büngner bands, nourishing damaged neurons and promoting axonal regeneration and myelination [17, 18]. The grafting of SCs was a successful strategy to repair peripheral nerve injury (PNI), by which nerve regeneration and functional recovery had been achieved in literatures [19–21]. Therefore, an improved SCs niche that could accelerate cell adhesion, migration and proliferation would further enhance cell expression and following nerve regeneration.

In this study, the influence of tag sequence on rhCol III was firstly investigated and compared *via* simulation and experiment methods. Then, the effects of rhCol III on the SCs behaviors including cell adhesion, spreading, migration, proliferation, neurotrophin secretion and gene expression were investigated *in vitro*. Additionally, the rhCol III-induced cellular responses in SCs were studied on a molecular level, concerning the integrin downstream kinases and structural proteins, such as focal adhesion kinase (FAK) and vinculin (VCL). The activation of appropriate motility signaling through integrins to regulate SCs behaviors was finally discussed.

## Materials and methods

### Materials

The lyophilized rhCol III obtained from Shanxi Jinbo Pharmaceutical Co., Ltd. was a rhCol with 16 tandem repeats of a functional domain (Gly483-Pro512) encoded by a gene segment of human collagen type III. The same rhCol III with a tag sequence (rhCol III-tag) at the N-terminal was prepared and studied at the same time. rhCol III samples were dissolved in Dulbecco’s Modified Eagle Medium (DMEM) or phosphate-buffered saline (PBS) to obtain the solutions at desired concentrations. All chemicals were used as received without further treatment unless otherwise stated.

### Molecular docking

The simplified amino acid sequences of rhCol III and rhCol III-tag for molecular docking analysis are listed in Table 1. Firstly, AF2 was used to predict the 3D structures of the proteins according to the amino acid sequences, and the optimal conformation was selected for the subsequent molecular docking analysis. Discovery Studio 2019 (DS, Accelrys, Biovia, USA) was used to remove ligands and water molecules from the crystal structure complex. Energy minimization of protein was conducted by using CHARMm force field, and the optimized tertiary structure of the crystal protein was finally achieved. Molecular self-dockings of rhCol III and rhCol III-tag were performed using the ZDOCK module analysis in DS. For each sequence, the predicted result with the highest ZDOCK score was selected for visual analysis.

**Table 1.** The amino acid sequences of rhCol III and rhCol III-tag for molecular docking

Type	Code	Amino acid sequence
rhCol III	C30	GERGAPGFRGPAGPNGIPGEKGPAGERGAP
rhCol III-tag	H10C30	HHHHHHHHHHGERGAPGFRGPAGPNGIPGEKGPAGERGAP
	T10C30	DLGTENLYFQGERGAPGFRGPAGPNGIPGEKGPAGERGAP

### Circular dichroism

rhCol III and rhCol III-tag were dissolved in PBS solution with a final concentration of 1 mg/ml and incubated overnight at 4°C. The ellipticity of different samples was measured, and the circular dichroism (CD) spectra were generated for structural analysis. The quartz cuvette was rinsed for 2–3 times using the sample solutions before the test respectively, and PBS solution was taken as the baseline calibration. The scanning range was 300–180 nm, the frequency bandwidth was 1 nm and the scanning speed was 50 nm/min. All sample tests were repeated three times.

### Cell culture

The rat SC line RSC96 (Chinese Academy of Sciences, Shanghai, China) was cultured in DMEM/high glucose medium (Gibco) with 10% fetal bovine serum (Gibco) and 1% Pen/Strep (P/S, Gibco) at 37°C and 5% CO_2_. Additionally, SCs were confirmed using the s-100β protein staining technique.

### Cell adhesion

The cell adhesion was evaluated according to a previously reported assay [6]. Briefly, rhCol III was dissolved in PBS to attain solutions with different concentrations (0, 0.01, 0.05, 0.1, 0.5 mg/ml), and then 100 μl of the solution was added to a 96-well plate and cultured at 4°C overnight. The nonspecific binding sites were blocked with 100 μl of 1% bovine serum albumin (BSA), followed by washing with PBS twice. 1 × 10^4^ SCs were added to each well and incubated for 60 min at 37°C and then washed with PBS twice. The attached cell numbers were tested by cell counting kit-8 (CCK-8).

### Cell immunofluorescence

According to the previously reported cell immunofluorescence method [8], 400 μl rhCol III solution (0.1 mg/ml) and PBS (as control) were added to a 24-well plate with sterile cell slides at 4°C overnight. SCs (2×10^4^ per well) were seeded on glass coverslips for 4 or 24 h. For immunofluorescence microscopy, cells were fixed in 4% paraformaldehyde (PFA, Sigma-Aldrich) for 20 min, washed and permeabilized with Triton X-100 (0.2% in PBS). The samples were blocked by 1% BSA for 30 min at room temperature. Fixed SCs were incubated with phalloidin (iFlour 488, MKbio) or primary antibodies: anti-s100β (Proteintech, 1:200), anti-VCL (CST, 1:200) and anti-p-paxillin (CST, 1:200), for 90 min at room temperature followed by goat anti-mouse antibody (Alexa Fluor 488, Abcam, 1:100) or goat anti-rabbit antibody (Alexa Fluor 647, Abcam, 1:100). 1 μM 4′,6-Diamidino-2-phenylindole (DAPI, BioLegend) was used to counterstain nuclei.

### Cell migration

5 × 10^5^ SCs were initially seeded in each well of 6-well plates. After reaching 90% confluence, the cells were cultured in DMEM without serum overnight. Then, a scratch was formed on the cell monolayer using a 200 μl pipette tip. Following the removal of cellular debris by PBS, DMEM with different concentrations of rhCol III (0, 0.01, 0.05, 0.1, 0.5 mg/ml) were added to the culture plates. Images of the cells were taken immediately after wounding and at time intervals of 12, 24, 36 and 48 h afterward. To acquire the data for further comparison and interpretation, reference points were put on the outside bottoms of plates to specify the scratch positions and widths, which were further calculated using image J software. For inhibitor experiments, VS-6063 (Beyotime) diluted to 10 μM by DMEM was added after cell scratching and washing by PBS. Inhibitors remained in the media during the experiment. The cell migration rate was calculated using the following equation:


$$\mathrm{Cell}\;\mathrm{migration}\;\mathrm{rate}=\left({D_{0}-D}_{X}\right)/D_{0}\times 100\%,$$

where $D_{0}$ was the initial scratch width, and $D_{X}$ was the scratch widths at set time intervals.

### Cell proliferation

For the cell proliferation assay, 5 × 10^3^ SCs were seeded in a 96-well culture plate and then cultured for 24 h and 48 h in DMEM with different concentrations of rhCol III (0, 0.01, 0.05, 0.1, 0.5 mg/ml) with six duplicates per group. The cell number was tested by CCK-8. The optical density 450 (OD 450) value was recorded to reflect cell proliferation ability.

### Quantitative real-time PCR

The total RNA of each sample was extracted using the TRIzol technique, closely adhering to the manufacturer's instructions (Invitrogen). Using a spectrophotometer, the concentration and purity of freshly extracted RNA were determined at 260 nm and calculated *via* the A260/280 ratio, respectively. Murine leukemia virus reverses transcriptase (TaKaRa) was utilized to synthesize the first-strand cDNA from 1 mg of RNA, which was then used for quantitative real-time polymerase chain reaction (qRT-PCR). Genes of brain-derived neurotrophic factor (BDNF) and nerve growth factor (NGF) were amplified and measured by qRT-PCR using SYBR Premix Ex Taq (TaKaRa) and LightCycler 96 (Roche). The 2^-ΔΔCt^ method was used to examine the relative expression levels, normalized with respect to the housekeeping gene GAPDH expression. The results were shown as a fold increase in comparison to the control group. The specific primers used in this experiment are shown in Table 2.

**Table 2.** Primers for qRT-PCR analysis

Gene	Forward	Reverse
GAPDH	GGTGGACCTCATGGCCTACA	GGGCCTCTCTCTTGCTCTCA
BDNF	GGAGGCTAAGTGGAGCTGACATAC	GTGCTTCCGAGCCTTCCTTTAGG
NGF	CCAAGCACTGGAACTCATACTGC	CTGCTGAGCACACACACGCAG

### Enzyme-linked immunosorbent assay

Commercially available enzyme-linked immunosorbent assay (ELISA) kits (Cloud-Clone) were applied to measure the concentrations of BDNF and NGF. Following a 24-h *in vitro* culture of SCs in DMEM with or without rhCol III (0.01, 0.05, 0.1 or 0.5 mg/ml), the secretions of BDNF and NGF in cell-cultured supernatant were detected following the manufacturer’s instructions with a necessary centrifugation of the supernatant to remove cellular waste.

### Western blot

The total protein of SCs expression was obtained using a protein extraction kit (PE001, SAB Biotech). The protein concentrations were measured using the Bradford assay (Beyotime). The protein samples were firstly heated with SDS-PAGE sample loading buffer (Beyotime) at 100°C for 5 min, and equal amounts of protein (20 μg) were then loaded onto a 7.5% SDS-polyacrylamide gel, separated by electrophoresis, and transferred to a polyvinylidene fluoride membrane (Millipore). The membranes were treated with the primary antibodies at 4°C overnight after antigen blocking. After three rounds of washing, the membranes were incubated at room temperature for an hour with HRP-labeled goat anti-rabbit IgG (Beyotime). The membranes were exposed *via* ChemiDoc XRS + (Bio-Rad) to detect the designated protein expression level. Using ImageJ software, the intensities of the bands (representing protein levels) were calculated. Target proteins were normalized using β-actin. The primary antibodies and concentrations were listed as follows: rabbit anti-β-actin antibody (Beyotime, 1:5000), rabbit anti-FAK antibody (CST, #71433, 1:1000), rabbit anti-pFAK (Tyr397) antibody (CST, #3283, 1:1000), rabbit anti-pFAK (Tyr576) antibody (CST, #3281, 1:1000), rabbit anti-vinculin antibody (CST, #13901, 1:1000).

### Statistical analysis

Data were expressed as the means ± standard deviation and were evaluated using a one-way analysis of variance with the Tukey HSD comparison test or using the independent-samples *t*-test. A statistically significant difference was determined to exist when the *P* value was <0.05.

## Results

### Influence of tag sequence on the advanced structure of rhCol III

**Figure 1.**
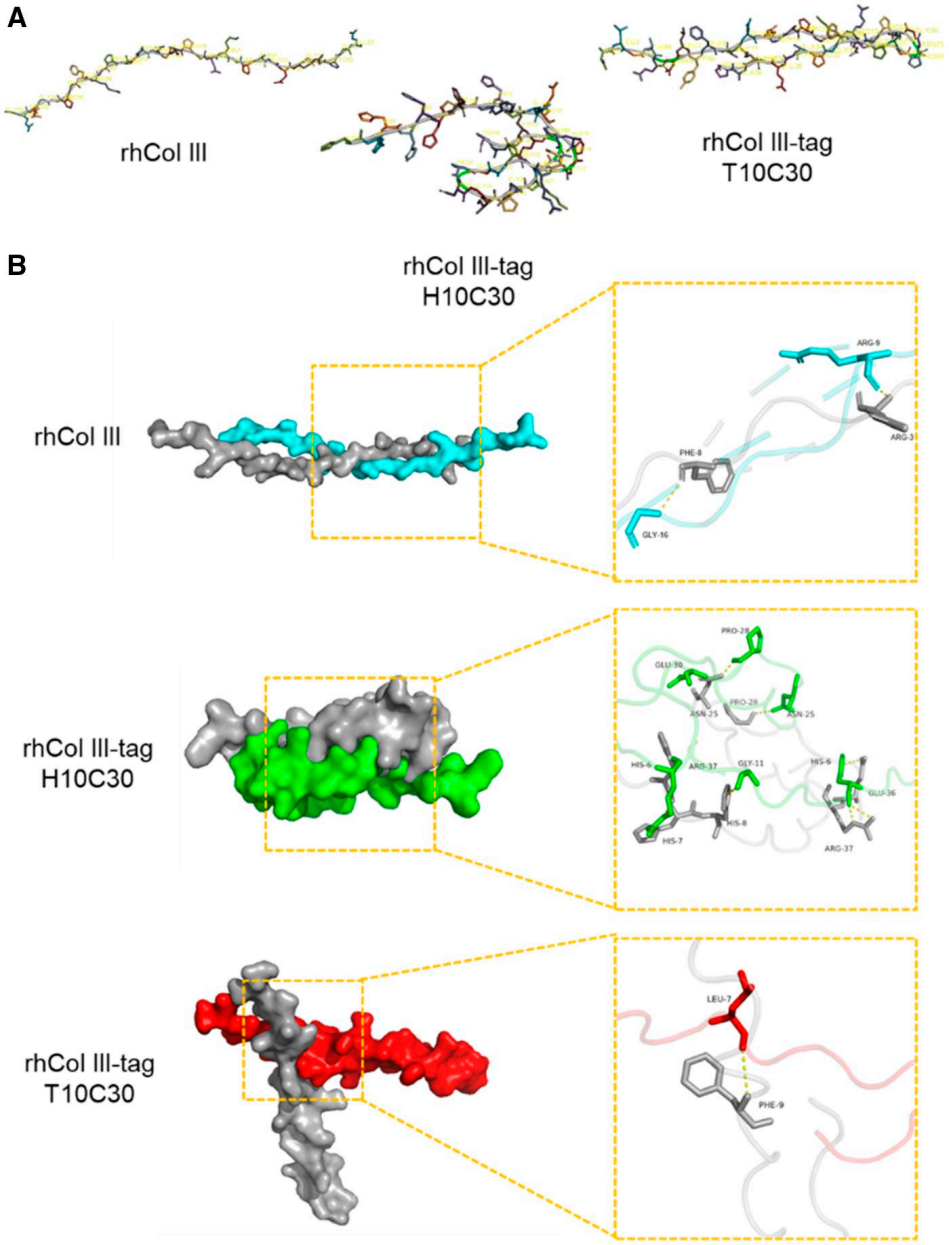
The 3D structure and binding sites of rhCol III-tag differ from rhCol III. (**A**) The 3D structure of rhCol III and rhCol III-tag (H10C30 and T10C30) predicted by Alphafold2. (**B**) The predicted molecular self-docking model with the highest ZDOCK. Yellow line: hydrogen bond.

To native collagen, the primary structure could be considered the fundament of collagen functional diversity, and the advanced structures including secondary and tertiary structures namely triple helix structures were determinants of cell–material interactions [22]. To recombinant collagen, the secondary or tertiary structure was crucial to the stability and function of the polypeptides when the primary structure was previously determined. In order to understand the influence of the artificially introduced tag sequence on the advanced structure of rhCol III, two representative tags with different amino acid sequences were designed and added to the N-terminal of rhCol III repeat unit, namely rhCol III-tag (H10C30, T10C30). The advanced structures of rhCol III and rhCol III-tag (H10C30, T10C30) predicted by AF2 are shown in Figure 1A. The results displayed that the introduction of additional amino acid sequences at the N-terminal could result in conformational changes in protein folding. The rhCol III molecule chain stretched with little helix structure of the backbone, while the continuous His tag protein H10C30 showed an obvious intramolecular folding structure, and the T10C30 presented a big turning structure with a reverse dihedral angle at Pro24-Asn25.

Based on the 3D structures of rhCol III and rhCol III-tag acquired by AF2 analysis, the molecular self-docking between two chains was simulated and analyzed, as shown in Figure 1B. In general, the associations of folded polypeptides at minimized energy were completely different among these rhCol III-based molecules, showing intermolecular twisting of rhCol III and entangling, partial overlapping of rhCol III-tag. The hydrogen bond numbers and distributions were further analyzed since it was one of the major intermolecular bonds that stabilized the advanced structures. As shown in Figure 1B, the hydrogen bonds were formed *via* intermolecular Arg9 and Phe8, Gly16 and Arg3 in rhCol III complex. In H10C30, the positive charged His residues were widely involved in generating intermolecular hydrogen bonds, including His6, His7 and His8, which created more folding and close entangling of the polypeptide chains. To T10C30, the intramolecular folding made the hydrogen bonds be formed only between residues with side groups exposed and stereospecific availability, like the Leu7 and Phe9 from the tag sequence. The results indicated that the addition of tag sequence would change the spatial conformation of rhCol III and the further generated hydrogen bonds would stabilize the advanced structures of polypeptide chains, which were significantly different from rhCol III without the tag sequence.

**Figure 2.**
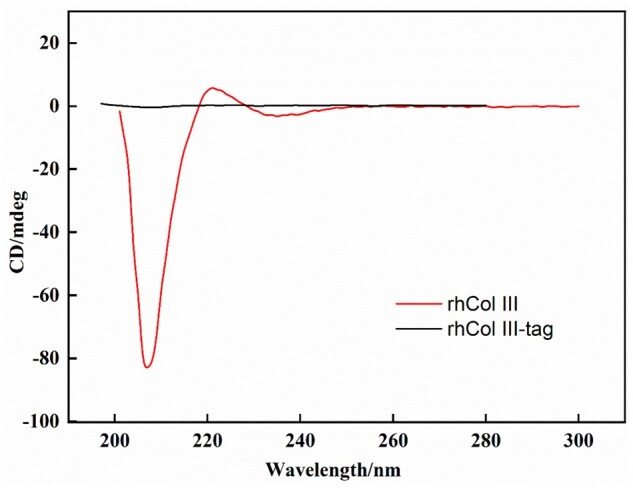
The CD spectra of rhCol III and rhCol III-tag.

The positive peak around 220 nm in CD spectra was one of the major characteristics of native collagen with a triple helix structure [23]. The disappearance or obvious shift of this featured peak could be considered that the triple helix structure was not preserved or denaturation of collagen. As shown in Figure 2, the rhCol III showed featured peaks in CD spectra as native collagen, which contained a strong negative peak near 207 nm and a positive peak near 220 nm. In contrast, there was no obvious peak could be found in CD spectra of rhCol III-tag, indicating the lack of protein-advanced structure. Thus, it could be inferred that introduction of tag sequence on rhCol III would lead to a significantly different advanced structure of polypeptide chains, which was consistent with the theoretical results.

### Influence of tag sequence on bioactivities of rhCol III

The SCs grown on a standard cell culture plastic could be identified by morphology which was typical bipolar or tripolar spindle shape as shown in Supplementary Figure S1 and immunostaining of s-100β antigen that was expressed by SCs and considered as characteristic of SCs [24]. SCs behaviors including cell adhesion, migration and proliferation were evaluated to understand the influence of tag sequence on the bioactivities of rhCol III.

**Figure 3.**
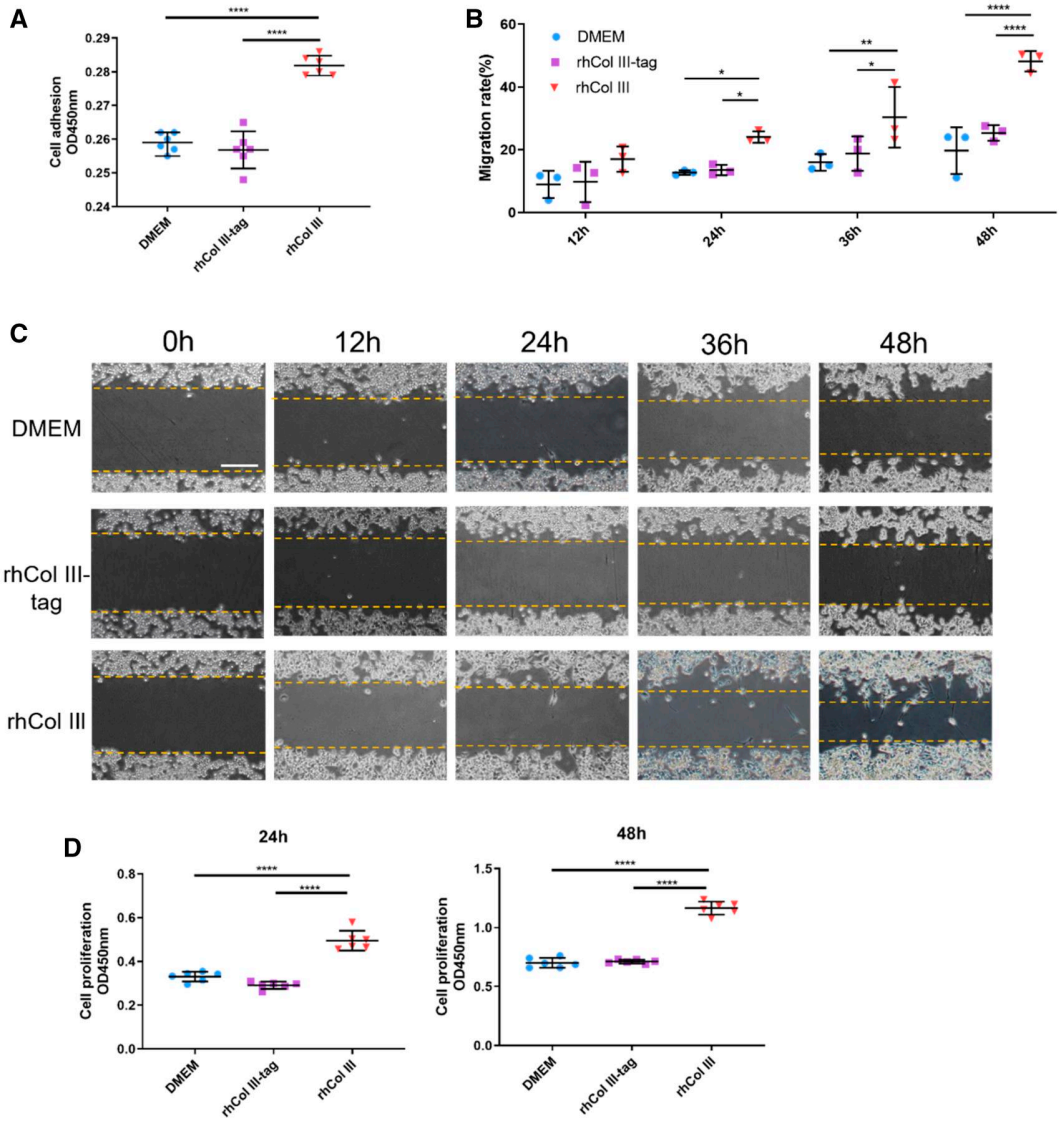
Comparison of the effects of rhCol III and rhCol III-tag on SCs adhesion, spreading, migration, and proliferation. (**A**) Cell adhesion assay of 1 h after SCs incubated. (**B** and **C**) Images of the scratch test and the quantitative analysis. Scale bar = 100 μm. (C) Cell proliferation assay for 24 and 48 h. *P < 0.05, **P < 0.01, ****P < 0.0001.

As shown in Figure 3A, the OD values of 0.1 mg/ml rhCol III group were significantly higher than those in blank control and rhCol III-tag groups, while the latter two suggested insignificant differences. It illustrated that rhCol III was beneficial to the adhesion of SCs, but the introduction of tag sequence would quench the cell–-rhCol III interactions to reduce cell adhesion. According to the images and statistical results shown in Figure 3B and C, the cell migration was significantly enhanced in the rhCol III group than those in the control and rhCol III-tag groups after co-cultured for 24 h, while the difference between control and rhCol III-tag was insignificant at all time intervals. The cell proliferation results (Figure 3D) further verified the positive effect of rhCol III and the influence of tag sequence on corresponding performance. Consequently, the tag sequence on rhCol III not only changed the advanced structure but also affected the interactions with cells which might be the most important characteristic of biomimetic biomaterials.

### Effect of rhCol III on SCs behaviors

**Figure 4.**
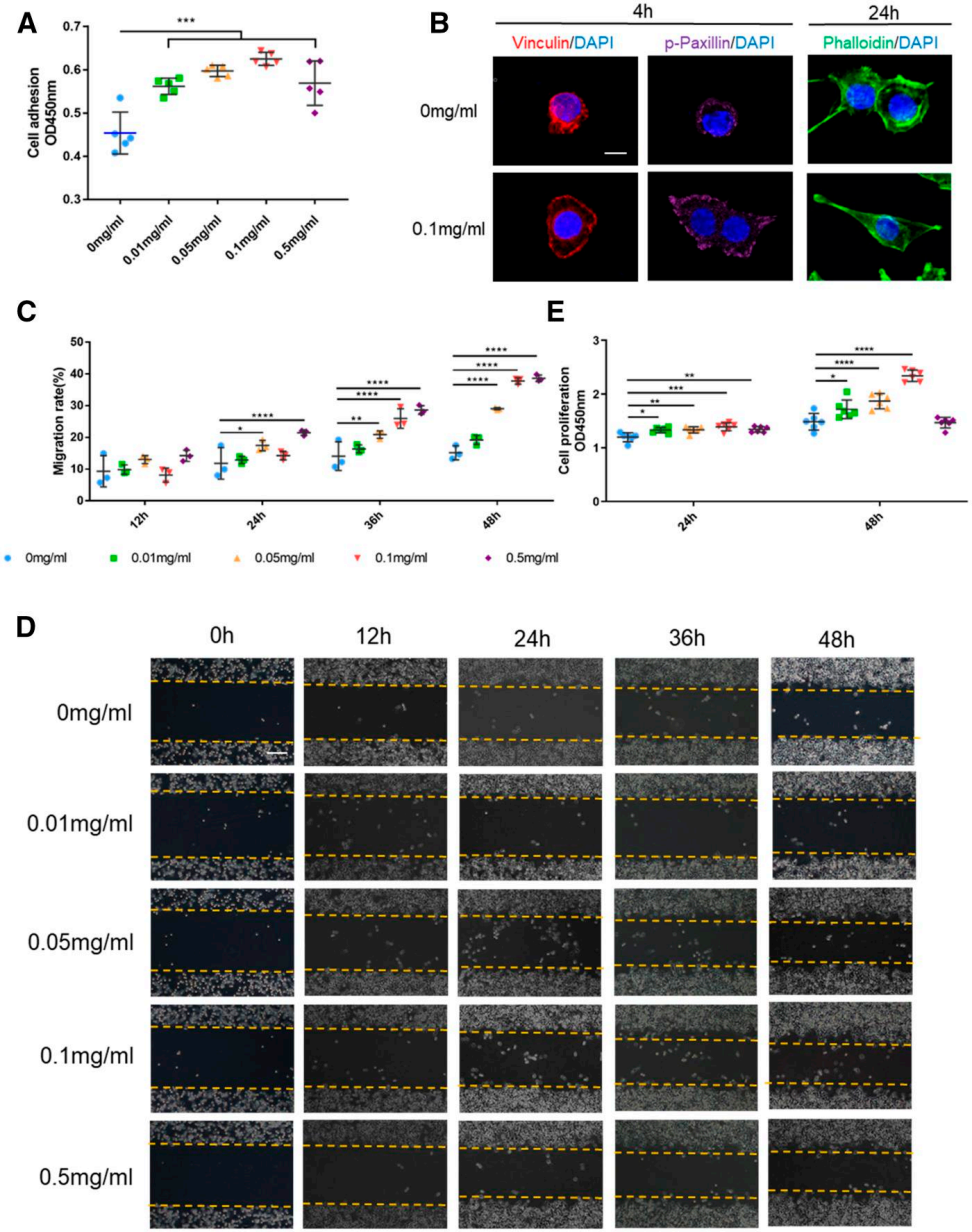
rhCol III Improved SCs adhesion, spreading, migration, and proliferation. (**A**) Cell adhesion assay of 1 h after SCs incubated. (**B**) Immunofluorescence staining on 4 h after SCs incubated and cytoskeleton staining on 24 h after SCs incubated. Red: vinculin. Purple: p-paxillin. Green: phalloidin. Blue: DAPI. Scale bar = 15 μm. (**C** and **D**) Images of scratch test and the quantitative analysis. Scale bar = 100 μm. (**E**) Cell proliferation assay for 24 and 48 h. *P < 0.05, **P < 0.01, ***P < 0.001, ****P < 0.0001.

rhCol III solutions with gradient concentrations were used to further explore the effect of rhCol III on SCs behaviors, including cell adhesion, spreading, migration and proliferation. As shown in Figure 4A, the SCs adhesions in all rhCol III groups were significantly higher than that in the control group, with the highest OD value attained in the 0.1 mg/ml group. Immunofluorescence staining results (Figure 4B) suggested that rhCol III promoted SCs spreading in the early cell adhesion period (first 4 h) [25]. Moreover, more and longer SCs filopodia were observed when co-cultured with 0.1 mg/ml rhCol III for 24 h.

The scratch assay and statistical results (Figure 4C and D) displayed that the migration rates of SCs in 0.05, 0.1 and 0.5 mg/ml rhCol III groups were significantly higher in comparison with the control at 24, 36 and 48 h post-exposure, while 0.5 mg/ml had the most obvious effect.

The CCK-8 assay was used to detect the proliferation of SCs, and the results are given in Figure 4E. The results showed that at 24 h after treatment, the OD 450 values of rhCol III groups (0.01, 0.05, 0.1, 0.5 mg/ml) were higher than that of the control group (*P*<0.05), indicating that rhCol III could promote SCs proliferation. At 48 h after treatment, the difference in cell proliferation between the rhCol III group (0.01, 0.05 and 0.1 mg/ml) and the control group was significantly increased. In addition, at both 24 and 48 h, the absorbance value of the 0.1 mg/ml group was higher than that of the other groups, indicating that 0.1 mg/ml rhCol III might be the most suitable concentration for promoting SCs proliferation at the current culture conditions.

### Effect of rhCol III on SCs functional expression

**Figure 5.**
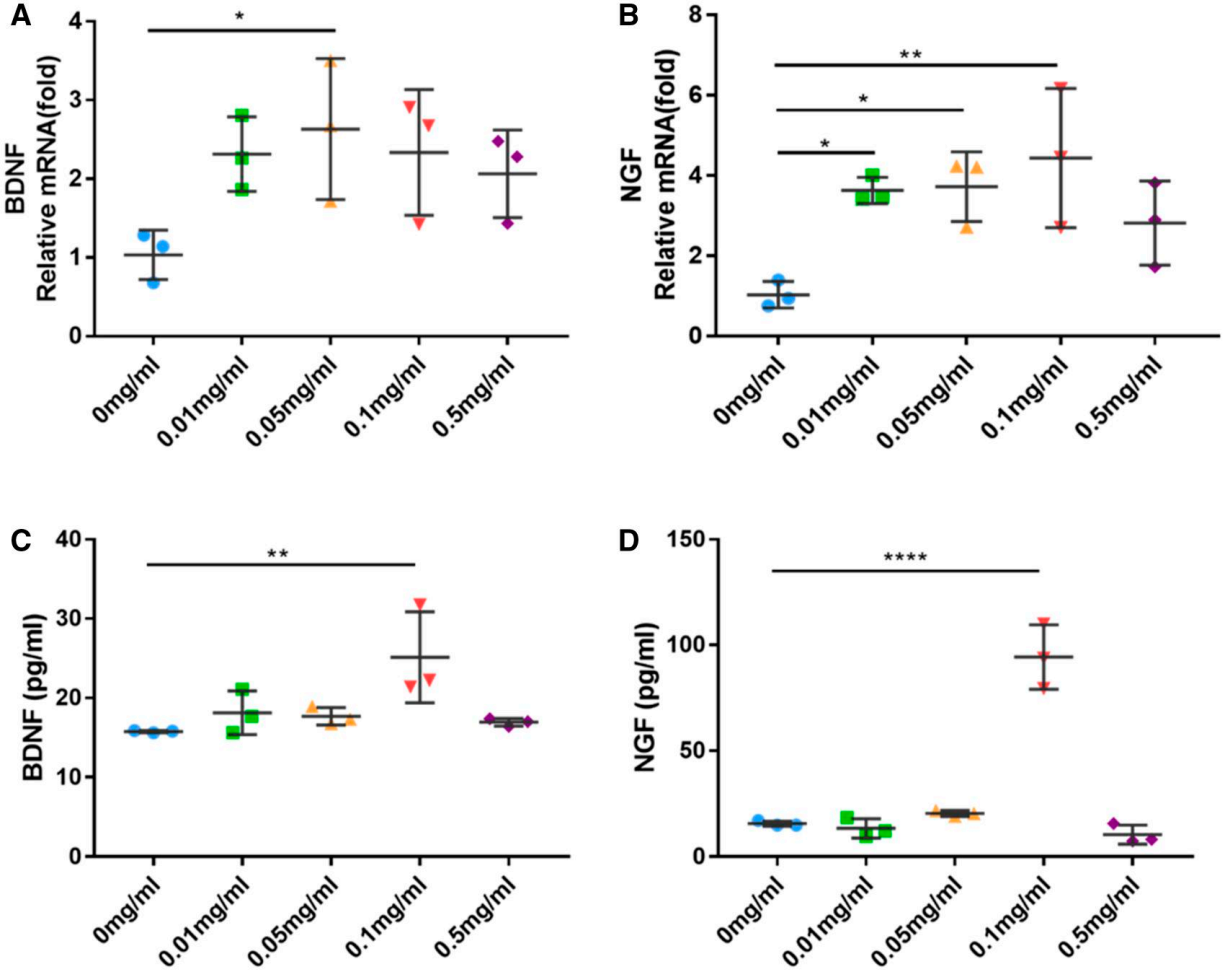
rhCol III Increased gene expression and secretion of BDNF and NGF. (**A**) Analysis of BDNF mRNA expression using qRT-PCR. (**B**) Analysis of NGF mRNA expression using qRT-PCR. (**C**) Analysis of BDNF protein expression using ELISA analysis. (**D**) Analysis of NGF protein expression using ELISA analysis. *P<0.05, **P < 0.01, ****P < 0.0001.

Since BDNF and NGF secreted by SCs could promote axon repair during nerve regeneration [26, 27], the expressions of these two distinctive genes were analyzed by qRT-PCR. The results revealed that the BDNF and NGF gene expression were increased in rhCol III groups. In the 0.05 mg/ml rhCol III group, the level of BDNF mRNA was increased to nearly 2.5-fold to that of the control group after 24 h of culture (Figure 5A). Figure 5B demonstrated that in the 0.01, 0.05 and 0.1 mg/ml rhCol III groups, the expression of the NGF gene was up-regulated to around 3.6-fold, 3.8-fold and 4-fold, respectively. Consistent with the gene expression results, SCs cultured with rhCol III led to a significant increase in neurotrophin secretion according to the results of ELISA assays. The concentrations of both BDNF and NGF increased largely in the 0.1 mg/ml rhCol III group, which were significantly higher than the control (Figure 5C and D, *P *<* *0.05).

### rhCol III promoted phosphorylation of FAK and expression of VCL

**Figure 6.**
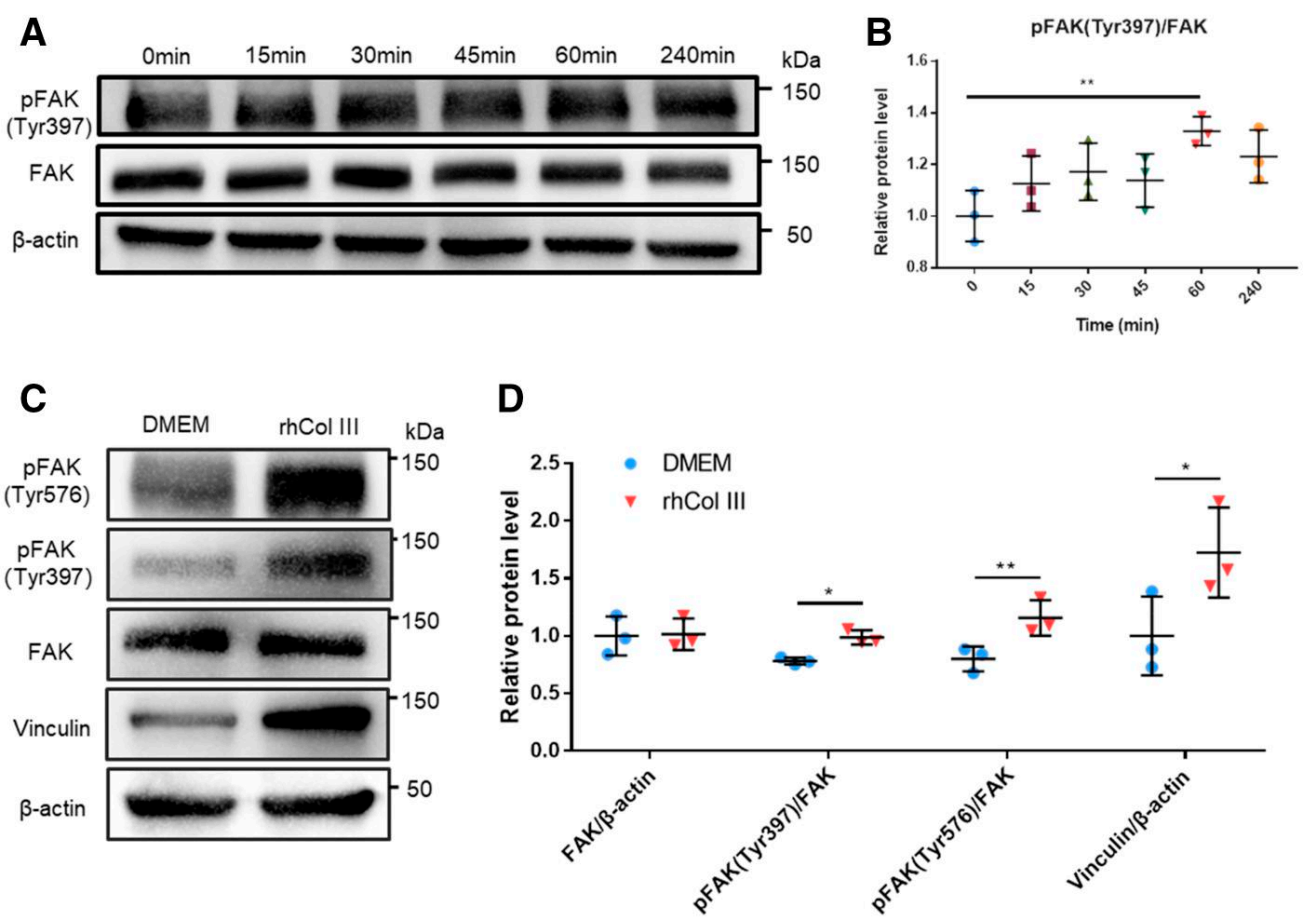
rhCol III promoted phosphorylation of FAK and expression of VCL. (**A**) Western blotting analysis of the expression of pFAK (Tyr397), FAK and β-actin in SCs treated within 0.1 mg/ml rhCol III at different times. (**B**) Quantitative analyses of pFAK (Tyr397)/FAK. (**C**) Western Blotting analysis of pFAK (Tyr576), pFAK (Tyr397), FAK, Vinculin and β-actin in SCs treated within 0.1 mg/ml rhCol III at 60 min. (**D)** Quantitative analyses of FAK/β-actin, Vinculin/β-actin, pFAK (Tyr397)/FAK and pFAK (Tyr576)/FAK. *P < 0.05, **P < 0.01.

Although the mechanism of cell–rhCol III interactions was not clearly understood, the specific domain recognized as integrin-binding sites in rhCol III was considered as the possible cause of bioactivity [28]. Therefore, the investigation of the downstream proteins of integrin activation was designed and conducted. FAK is a cytoplasmic tyrosine kinase that is primarily mediated by integrin signaling, while the activation of FAK was carried out firstly *via* auto-phosphorylation at Tyr397 and subsequently *via* phosphorylation at Tyr576 [29, 30]. Compared with the control group, the phosphorylation of FAK (pFAK) at the Tyr397 increased with rhCol III treatment during the test period of 240 min, and the highest expression level was observed at 60 min (Figure 6A and B). Moreover, the subsequently significant (*P *<* *0.05) increase of pFAK at Tyr576 was detected when co-cultured with 0.1 mg/ml rhCol III for 60 min, while the FAK expression was insignificantly changed compared with the control group (Figure 6C and D), synchronously. VCL is a cytoplasmic actin-binding protein enriched at cell–matrix adhesion and is present in all force-bearing cellular junctions, which is involved in cell adhesion and migration [31–33]. Figure 6C and D shows that the expression of VCL in the rhCol III group was up-regulated to 1.7-fold that that in the control group. Therefore, the results of western blotting demonstrated that the rhCol III promoted SCs adhesion and downstream cell expressions *via* the phosphorylation of FAK and expression of VCL.

### Verification of the promotion of rhCol III on phosphorylation of FAK

**Figure 7.**
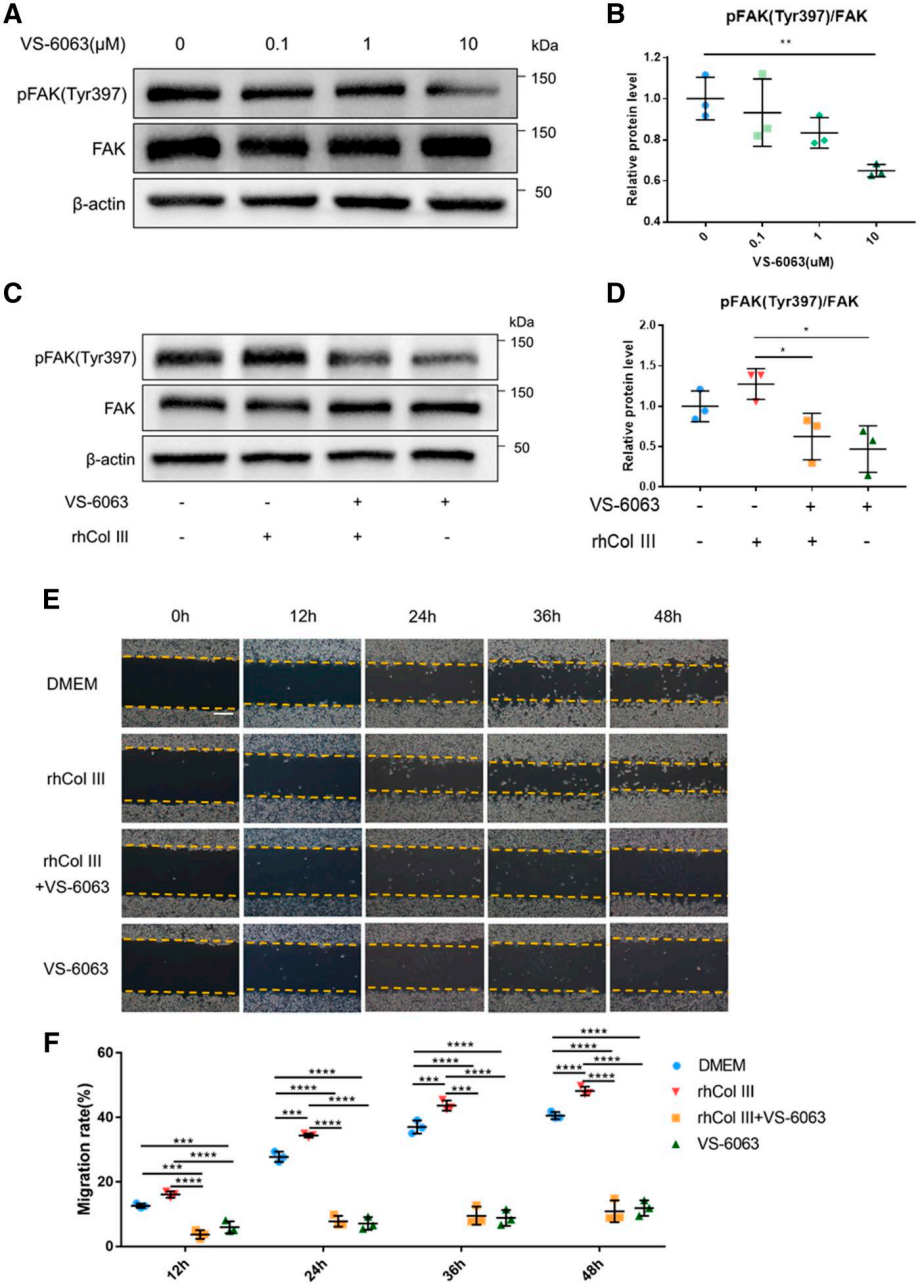
Inhibition of pFAK (Tyr397) decreased SCs migration. (**A**) Western blotting analysis of the expression of pFAK (Tyr397), FAK and β-actin in SCs treated with different concentrations of VS-6063. (**B**) Quantitative analyses of pFAK (Tyr397)/FAK. (**C**) Western blotting analysis of the expression of pFAK (Tyr397), FAK and β-actin in SCs treated with 10 μM VS-6063 and/or 0.1 mg/ml rhCol III. (**D**) Quantitative analyses of pFAK (Tyr397)/FAK. (**E**, **F**) Images of scratch test and the quantitative analysis. Scale bar = 100 μm. *P < 0.05, **P < 0.01, ***P < 0.001, ****P < 0.0001.

VS-6063 is an inhibitor of FAK phosphorylation and was applied to verify the promotion effect of rhCol III on the phosphorylation of FAK. The western blotting results showed that the expression of pFAK (Tyr397) was statistically significantly inhibited by 10 μM VS-6063 (Figure 7A and B). After treatment with 0.1 mg/ml rhCol III, the phosphorylation level of FAK at Tyr397 increased, while the expression of pFAK at Tyr397 was inhibited by the addition of 10 μM VS-6063 (Figure 7C and D). The further cell migration assay with and without rhCol III and inhibitor showed that 0.1 mg/ml rhCol III could promote SCs migration, while 10 μM VS-6063 significantly attenuated this effect (Figure 7E and F). Considering the results of western blotting and cell migration assay together, it suggested that the activation of FAK was a main pathway by which rhCol III could modulate SC migration.

## Discussion

rhCol could be considered a novel type of biomimetic biomaterial that was inspired by native collagen [2, 4]. The current research using rhCol as the starting material for implantable biomaterials and medical devices has shown great potential in tissue regeneration with desired biosafety and efficiency [7, 8, 34, 35]. However, the influence of the primary and advanced structure on rhCol properties still needed systematic investigation, while the mechanism of cell–rhCol interactions was not fully understood. Therefore, using rhCol III as an example in this study, it was greatly significant to realize the influence of tag sequence on both structures and bioactivities and preliminarily clarify the pathway of cell–rhCol interactions.

For a long time, homology modeling had been used to construct the 3D structure of the protein (recombinant collagen), especially when there was a specific primary sequence and the known 3D structures of homologous proteins (native collagen) [36–38]. Currently, AlphaFold2 could predict 3D structures of proteins based on amino acid sequences with atomic-level accuracy [12]. To understand the influence of tag sequence on recombinant collagen with a simplified model, two representative tag sequences were designed and analyzed by AF2 in this study, because both of them had been successfully applied in the high-yielding purification of recombinant protein mass-production which showed superiorities of high-efficiency labeling and controllable removal. The H10C30 represented the consecutive six-histidine-based fusion protein which was widely applied in multiple recombinant expression system [39, 40]. T10C30 exemplified the tag protein with the high specificity of TEV protease, and it was mainly introduced into the target protein to achieve mass-scale-specific purification [41, 42]. The prediction results of rhCol III with these different tag sequences showed obviously different protein conformations from that of rhCol III probably because of the change of local charges and formation of intramolecular hydrogen bonds. The amino acid sequence in C30 was completely the same as that in human collagen type III of Gly483-Pro512, which featured with paired existence of most acidic (negatively charged) and basic (positively charged) amino acid residues, and evenly distribution of non-polar and polar (uncharged) amino acid residues, leading to a charge balanced and unfolded molecular chain. This stretched conformation of rhCol III was beneficial to the generation of further advanced structures, which could be testified by the molecular docking analysis. The intermolecular hydrogen bonds were mainly generated with the involvement of the only un-paired arginine with positive charge in the sequence of rhCol III, which should be important to stabilize the rhCol III aggregates. In contrast, the introduction of tag sequences would destroy the charge balance and distribution, no matter the consecutive histidine sequence or the flexible tag which were positive charged and negative charged, respectively [43]. The residues in tag sequence with un-paired charges might attract the approaching of oppositely charged residues in the rhCol III and new intramolecular and intermolecular hydrogen bonds were formed to stabilize the protein conformation from further molecule assembly. Also, the traditional structural characterization of rhCol III and rhCol III-tag demonstrated the introduction of tag sequence would disturb the forming of rhCol III advanced structures, which supported that the homology modeling was effective in characterize the structures of rhCol III.

It was universally accepted that the protein structures determined the properties and performances. Since the design and development strategy of rhCol was based on the utilization of functional domains in native human collagen, the modulating function on cell behaviors was one of the major objectives that would show the great influence of rhCol performances in the potential application. The results of cell adhesion, migration and proliferation assays verified that the design and development strategy of rhCol III was successful as reported in the literature and suggested that the introduced tag sequence showed great influence on its bioactivity which was consistent with the results of structural investigation discussed previously. Although the direct relationship between protein structure and bioactivity property was not clearly understood, it was necessary to realize that the molecular conformation and further intermolecular assembly would determine the cell–matrix communication and downstream biological response *via* many different pathways. Consequently, the formation and maintenance of the biomimetic structures should be considered in the development and characterization of recombinant collagen-based biomaterials or medical devices.

The following co-culture of rhCol III and SCs was intended to understand the effect and possible mechanism using peripheral nerve regeneration as an application scenario. According to the results of *in vitro* cell culture with different concentrations of rhCol III, an obvious cell behavior modulation was achieved at 0.1 mg/ml rhCol III at the given culture conditions. Especially, in the early cell adhesion period (first 4 h), rhCol III influenced SCs to show more filopodia, which were finger-like protrusions that contain bundles of linear F-actin, and to provide a better probing of micro-environmental cues and directionality of migrating cells [44]. The scratch assay further confirmed that rhCol III was conducive to maintain the persistence of cell migration. Even though the effect of rhCol III on SCs was approved, which agreed with the results when rhCol III co-cultured with 3T3 cell line [6], human uterosacral ligament fibroblasts (HULFs) [34], endometrium stromal cells [45], keratinocytes [46], endothelial cells and fibroblasts [47], the optimized rhCol III concentration might be varied in different cell models. There were many reasons for this variation, and a possible explanation was that the rhCol III would self-assemble into aggregates with different diameters and lengths at different concentrations. Generally, a higher concentration would lead to a thicker and longer aggregation of polypeptide chains, which are possessed with distinctive cell recognition sites. While the adhesion receptors (integrins) on different cell surfaces were quantitatively unequal and inconsistently distributed, the cell–rhCol III interactions would occur at high incidence when the suitable aggregations were achieved under certain conditions. In this study, the concentration of 0.1 mg/ml rhCol III might be the optimized condition for self-assembly and interaction with SCs.

Following the adequate adhesion, migration and proliferation of SCs, the functional expression of SCs was also essential for the peripheral nerve regeneration after injury [48]. Since BDNF and NGF were important neurotrophins secreted by SCs, measurements of these two factors could enhance the reliability of using SCs as an *in vitro* model of peripheral nerve regeneration. By binding to a particular transmembrane tyrosine kinase receptor, BDNF could initiate neuroprotective effect which was crucial in the nervous system [26]. As the earliest and best-studied member of the neurotrophin family, NGF could regulate the differentiation and survival of neurons in both the central and peripheral nervous systems [27]. The current qRT-PCR and ELISA results accomplished in this study demonstrated a noticeable elevation of BDNF and NGF gene expression and protein secretion of SCs cultured with rhCol III. Although Zhang et al. speculated that actin cytoskeletal changes might mediate the secretion of neurotrophins by SCs [49], the underlying cause of up-regulated expression of BDNF and NGF was unclear. It might be related to the enhanced cell adhesion and migration which was also involved with the alteration of the cytoskeleton and mechanical transmitting in some degree. Nevertheless, more studies still need to be conducted to understand the mechanism of rhCol III on the regulation of neurotrophins.

As a non-receptor tyrosine kinase, FAK was the earliest identified and one of the most prominent signaling molecules which coupled with integrins to regulate cell adhesion and migration. Upon integrin-mediated cell adhesion on ECM, full activation of FAK was progressively achieved by phosphorylation of Tyr397, Tyr527 and Tyr576, etc. and further binding and activation of Src family kinase [29]. The mutually activated FAK/Src complex then initiated multiple downstream signaling pathways to regulate different cellular functions. The up-regulation results of pFAK acquired in this study demonstrated a higher activation degree of FAK that the integrin-mediated SCs adhesion was promoted when co-cultured with 0.1 mg/mL rhCol III. Li et al. also found that rhCol III promoted HULFs adhesion and migration by upregulating the FAK signaling pathway *in vitro* and *in vivo* [34]. Meanwhile, the VCL expression was significantly up-regulated in the rhCol III group according to the western blotting results in this study. As an important cytoplasmic protein, VCL could directly bind to actin, stimulate actin polymerization and recruit actin remodeling proteins, which was critical in cell–matrix adhesion [32, 33].

In terms of cell migration, it was considered a dynamic and multistep process including protrusion of the leading edge, adhesion to the substratum, generation of traction forces to propel the cell forward and breaking of older adhesions by tail retraction and detachment [29]. In these steps, both FAK and VCL played important roles. Several downstream signaling pathways could be activated through FAK in mediating the promotion of cell migration, such as the association and phosphorylation of p130Cas or paxillin by the FAK/Src complex [50, 51], interactions with PI3K [52], and effects on the Rho subfamily of small GTPases [53]. In regard to VCL, it was considered to be localized to the nascent adhesion in the leading edge of the cell [54, 55] and regulate the assembly of talin to stabilize the focal adhesions and promote integrin clustering [56]. Considering the results of cell migration assays together with the western blotting tests with and without inhibitor VS-6063, rhCol III would further accelerate SCs migration with activation of FAK and expression of VCL following the promoted SCs adhesion. Although the primary structure in rhCol III which included integrin-binding sites like Glu-Arg-Gly triplet might initialize the cell–rhCol III interactions [6], the fully molecular mechanism of rhCol III on SCs still needs further investigation.

It should be noticed that both rhCol III and rhCol III-tag for theoretical advanced structure analysis were simplified because of the limitation of available hardware and technology, and the representative advanced structure characterization was utilized as well. Even though the cytocompatibility was confirmed and regulation effects on SCs behaviors together with possible signaling pathways were preliminarily investigated, the biological performance of rhCol III still needs exploration *in vivo* in the future. Substantial and additional neural electrophysiological research and other efficacy verifications utilizing animal models must be carried out to evaluate the peripheral nerve regeneration ability of rhCol III.

## Conclusion

The influence of tag sequence on rhCol III and the effect of rhCol III on SCs was investigated and discussed in this study. Theoretically and practically, the introduction of tag sequence showed great influence on rhCol. Not only the advanced structure of the polypeptide chains would be altered with the tag sequence, but also the cell–material interactions would be completely changed. The *in vitro* cell culture results indicated rhCol III could promote SCs adhesion, spreading, migration and proliferation, and contributed to the increase in the gene expression and secretion of BDNF and NGF. As suggested by the western blotting results with and without inhibitor, rhCol III promoted SCs adhesion and migration partly through the FAK phosphorylation and VCL expression. The current findings contributed to a better knowledge of the interactions between rhCol III and SCs, and further offered a theoretical and experimental foundation for the development of rhCol III-based medical devices and clinical management of PNI.

## Supplementary Material

rbad089_Supplementary_Data
