# Supplementary material for: The influence of tag sequence on recombinant humanized collagen (rhCol) and the evaluation of rhCol on Schwann cell behaviors
Source: Regen Biomater. 2023 Oct 16;10:rbad089. doi: 10.1093/rb/rbad089 (PMC10676520; doi:10.1093/rb/rbad089)
Supplement: rbad089_Supplementary_Data [file rbad089_supplementary_data.docx]

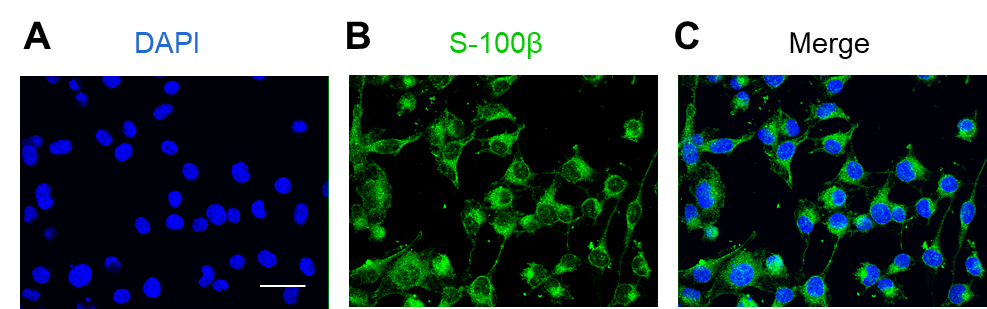


**Supplement Figure 1. S-100β immunostaining of Schwann cells**

All cells were stained homogenously by green fluorescent dye conjugated with antibody. (A) Nuclei stained with DAPI. (B) s-100β protein stained green. (C) Merged image. Scale bar = 30 μm.
